# Toll-Like Receptor 4 Inhibition Improves Oxidative Stress and Mitochondrial Health in Isoproterenol-Induced Cardiac Hypertrophy in Rats

**DOI:** 10.3389/fimmu.2017.00719

**Published:** 2017-06-22

**Authors:** Parmeshwar B. Katare, Pankaj K. Bagul, Amit K. Dinda, Sanjay K. Banerjee

**Affiliations:** ^1^Drug Discovery Research Center (DDRC), Translational Health Science and Technology Institute (THSTI), Faridabad, India; ^2^Department of Pathology, All India Institute of Medical Sciences (AIIMS), New Delhi, India

**Keywords:** cardiac hypertrophy, isoproterenol, toll-like receptor 4, lipopolysaccharide, RS-LPS, oxidative phosphorylation

## Abstract

**Background:**

Inflammation remains a crucial factor for progression of cardiac diseases and cardiac hypertrophy remains an important cause of cardiac failure over all age groups. As a key regulator of inflammation, toll-like receptor 4 (TLR4) plays an important role in pathogenesis of cardiac diseases. Being an important regulator of innate immunity, the precise pathway of TLR4-mediated cardiac complications is yet to be established. Therefore, the primary objective of the present study was to find the role of TLR4 in cardiac hypertrophy and the molecular mechanism thereof.

**Methods:**

Cardiac hypertrophy was induced with administration of isoproterenol (5 mg/kg/day, sc). TLR4 receptor inhibitor RS-LPS (lipopolysaccharide from the photosynthetic bacterium *Rhodobacter sphaeroides*; 5 μg/day) and agonist lipopolysaccharide (LPS) (from *Escherichia coli*; 3.12 μg/day) were administered through osmotic pump along with isoproterenol. Cardiac hypertrophy as well as oxidative stress and mitochondrial parameters were evaluated.

**Results:**

Cardiac hypertrophy was confirmed with increased heart weight/body weight ratio as well as assessment of hypertrophic markers in heart. There was a marked increase in the TLR4 expression and oxidative stress along with mitochondrial dysfunction in ISO group. TLR4 inhibition significantly decreased heart weight/body weight ratio and ANP, collagen, and β-MHC expression and restored the disturbed cellular antioxidant flux. The mitochondrial perturbations that were observed in hypertrophy heart was normalized after administration of TLR4 inhibitor but not with the agonist. TLR4 agonism further exaggerated the oxidative stress in heart and hence accelerated the disease development and progression.

**Conclusion:**

Our data show that increased TLR4 ligand pool in cardiac hypertrophy may exaggerate the disease progression. However, inhibition of TLR4 attenuated cardiac hypertrophy through reduced cardiac redox imbalance and mitochondrial dysfunction.

## Introduction

The ability of the myocardium to successfully adapt cellular stress ultimately determines whether the heart will maintain its preserved function or decompensate and fail. Despite the importance of the myocardial response to environmental stress, very little is known with respect to the biochemical mechanisms that are responsible for mediating and integrating the stress response in the heart (1). Cardiac hypertrophy is a result of an adaptive response to pressure or volume stress. Hypertrophic growth accompanies many forms of heart disease, including ischemic disease, hypertension, heart failure, and valvular disease (2). Despite the application of state-of-the art therapy, mortality remains high at two times at 5 years after detection of the disease. Therefore, we need to seek better strategies for the treatment of cardiac hypertrophy (3–5).

Inflammation is a common phenomenon observed in case of cardiac hypertrophy. This persistent inflammation of myocardium may play a key role in disease progression. However, the underlying mechanism remains unclear. This sterile inflammation in heart is mainly regulated by toll-like receptors (TLRs). TLRs are the pattern recognition receptors (PRRs), which play an important role in innate immunity by recognizing pathogen-associated molecular patterns and damage-associated molecular patterns (DAMPs) (6). There are 13 different TLRs in mammalian cell, among which toll-like receptor 4 (TLR4) is the most studied and is a central mediator of sterile inflammation (7). TLR4 is expressed in variety of cells including cardiomyocytes (8). TLR4-mediated signaling activates NF-κB, which regulates inflammatory and immune responses, cell growth, cell survival, and cell death (9).

Apart from inflammation, TLR4 is also involved in impaired cardiac function during sepsis-induced heart failure (10, 11). The role of TLR4 activation is not only limited to septic cardiomyopathy but also plays a significant role in other cardiac diseases. It has been observed that TLR4 knockout mice are protected from ischemia reperfusion injury (12). TLR4 has been known to be involved in modulation of myocyte contractility, left ventricular hypertrophy, and ischemia reperfusion injury. Increased TLR4 expression has been reported in the myocardium from patients with heart failure and ischemia (13, 14). Inhibition of Myd88, a downstream mediator of TLR4, leads to reduced left ventricular remodeling and improved cardiac function after aortic banding (15). Studies performed by Ha et al. (16) and Ehrentraut et al. (17) had shown the protection against pressure overload cardiac hypertrophy in TLR4-deficient mice by decreased NFκB and cardiac inflammation. Similarly, TLR4 inhibitor “Iritoran” has protective effect against cardiac hypertrophy through PI3K/Akt/mTOR axis (18).

Two recent studies also have shown the improvement in cardiac function after central blockade (brain) of TLR4 through downregulation of myocardial inflammation and sympathetic activity (19, 20). All of these studies were focused to find the effect of TLR4 deficiency or inhibition on cardiac inflammation. However, in the present study, we have analyzed the effect of TLR4 modulation, i.e., both TLR4 agonist and antagonist on isoproterenol-induced rat cardiac hypertrophy focusing more on mitochondrial dysfunction.

Mitochondria are very important cellular organelles of cardiomyocytes, which need constant supply of high level of ATP for contraction. Cardiac hypertrophy is a serious health problem and progress slowly to failure heart. Mitochondrial dysfunction is a crucial event during the transition from hypertrophy to failure. It has been reported that LPS causes mitochondrial dysfunction through TLR4 activation (10, 21). Activation of TLR4-mediated signaling pathway may lead to release of many cytokines responsible for local as well as systemic inflammation. Therefore, we hypothesized that pharmacological modulation of TLR4 mitigates cardiac hypertrophy in rats, *via* attenuation of oxidative stress and mitochondrial dysfunction. The study proposes a strong rationale to investigate the potential application of TLR4 inhibitor and agonist in the treatment of cardiac hypertrophy and mitochondrial dysfunction.

## Materials and Methods

### Animal Study

All experiments involving animals were undertaken with the approval of Institutional Animal Ethical Committee of Indian Institute of Chemical Technology, Hyderabad. Male Sprague-Dawley rats weighing 200–250 g were purchased from Tina lab, Hyderabad, India. Animals were housed in BIOSAFE, an animal quarantine facility of Indian Institute of Chemical Technology, Hyderabad. Animals were maintained at temperature 22 ± 2°C with relative humidity of 40 ± 15% and 12-h dark/light cycle throughout the experiment. Animals had a free access to water and diet.

### Drug Solution Preparation and Dosing

Lipopolysaccharide (LPS) from *Escherichia coli* 0111:B4 strain (Invivogen) a potent TLR4 agonist and LPS from the photosynthetic bacterium *Rhodobacter sphaeroides* (RS-LPS) (Invivogen) a potent TLR4 inhibitor were used for TLR4 modulation (22). Solutions of modulators were prepared in pyrogen-free saline water and filled in the alzet pumps for sustained release. Isoproterenol (Sigma) solution was prepared in 0.4 mM ascorbate buffer immediately before dosing.

### Induction of Cardiac Hypertrophy in Rat and Treatment Schedule

Animals were randomly divided into four groups: control group (CON) was administered pyrogen-free saline through s.c. route. Hypertrophy group (ISO) was administered isoproterenol 5 mg/kg/day s.c. route (23). In addition, pyrogen-free saline-filled alzet pump was implanted subcutaneously on dorsal side in both the groups. Hypertrophy + TLR4 agonist group (LPS + ISO) was administered 3.12 μg/day of LPS through alzet pump along with isoproterenol (5 mg/kg/day, sc). Hypertrophy + TLR4 antagonist group (RS + ISO) was administered 5 μg/day of RS-LPS through alzet pump along with isoproterenol (5 mg/kg/day, sc). The whole treatment schedule was followed for a period of 14 days (*N* = 10). Animals were anesthetized using a mixture of ketamine (75 mg/kg, IP) and xylazine (5 mg/kg, IP) for surgical insertion of alzet pump. Body weight gain was monitored during the study period. At the end of study, all animals were sacrificed with high dose of anesthesia, heart was collected, and stored in −80°C or 4% formalin for downstream analysis.

### Heart Weight and Body Weight Ratio

After 14 days of experiment, rats were sacrificed. Heart was removed and washed in freshly prepared phosphate buffer solution. Heart was dried on a tissue paper and weighed. Body weight of all animals was measured just before sacrificing the animals. Heart weight/body weight ratio (milligrams per gram) was used for measuring cardiac hypertrophy as described earlier (24).

### Preparation of Heart Tissue Homogenate

Rat heart tissue homogenate was prepared by homogenizing 100 mg of heart tissue in 2 ml of 0.05 M phosphate buffer (pH-7.4) and centrifuging at 15,000 rpm for 30 min at 4°C. The resulting supernatant was stored at −80°C for further analysis.

### TLR4 ELISA Protein Estimation

Toll-like receptor 4 protein was analyzed using ELISA assay kit from Wuhan USCN, USA. Heart tissue homogenate was used to determine the TLR4 protein expression in rat heart (*N* = 4) and estimated following manufacturers protocol. The TLR4 protein was expressed as picograms per microgram of protein.

### Estimation of Antioxidant Parameters

Tissue homogenate was used for the estimation of thiobarbituric acid reactive substances (TBARS). Heart tissue homogenate supernatant was used to estimate reduced glutathione (GSH), glutathione peroxidase (GPx), glutathione reductase (GR), superoxide dismutase (SOD), reactive oxygen species (ROS), and catalase (CAT). TBARS (25) and ROS (26) were measured as markers of lipid peroxidation while GSH, SOD, CAT, GPx, and GR (27) were estimated as levels of endogenous antioxidants as described before (28).

### Isolation of Mitochondria

Mitochondria were isolated from equal weight of heart tissues using mitochondria isolation kit (Pierce, Thermo Scientific, Cat No: 89801). Briefly, heart tissue was cut into small pieces and homogenized using dounce homogenizer and the homogenate was then treated according to manufacturer’s protocol. The resultant mitochondrial pellet was suspended in MTP buffer containing 110 mM mannitol, 60 mM Tris HCL, 60 mM potassium chloride, 10 mM dibasic potassium phosphate, and 0.5 mM EDTA, pH-7.4. Mitochondrial purity and integrity was confirmed using Mito Tracker as described before (29).

### Mitochondrial Respiratory Chain Complex Activity in Heart

The specific enzymatic activity of mitochondrial electron transport chain (ETC) complex I (NADH-ubiquinone oxidoreductase) and complex II (succinate-ubiquinone oxidoreductase) were measured in the isolated mitochondria from heart as previously described (30). β-hydroxyacyl CoA dehydrogenase activity, an important enzyme for beta oxidation and citrate synthase activity, an important enzyme of TCA cycle, were measured according to the protocol described previously (31, 32).

### Electrocardiogram Recording

ECG was measured on 13th day of the experiment. Animals were anesthetized using ketamine (75 mg/kg, IP) and xylazine (5 mg/kg, IP) in a mixture and kept in supine position on homeothermic blanket to maintain the body temperature throughout the experiment. ECG was measured using PowerLab with LabChart software as described before (28) (*N* = 4). Heart rate data were used to measure the tachycardia.

### Histopathology

Myocardial tissue was fixed in 4% neutral buffered formalin for 48 h. Fixed tissue was processed routinely and embedded in paraffin. Paraffin sections (5 µm) were cut and mounted on glass slides and stained with Hematoxylin and Eosin (H&E) and Masson’s trichrome stains and examined under a light microscope as described previously (27, 33). Cardiomyocytes cell size was analyzed using H&E-stained sections and cardiac fibrosis was analyzed using Masson’s trichrome stain with the help of ImageJ software as described before (24, 34).

### Gene Expression Profiling

RNA was isolated from heart tissue of all groups (*N* = 4) using TRIzol reagent (Sigma-Aldrich) following manufacturers protocol. Quantification and quality assessment of RNA was carried out with Nano Drop spectrophotometer (Thermo Scientific) and running on 1% agarose gel prepared in DEPC-treated TBE buffer. The extracted RNA was stored in −80°C for future use. RNA was treated with DNase before cDNA synthesis. cDNA was synthesized using 2 µg RNA with superscript-III reverse transcriptase (Takara, USA). Real-time polymerase chain reaction was carried out using Step One plus (Applied Biosystems Inc., USA) and SYBR Green mix (Takara, USA). The data were normalized to the expression of reference gene, *ribosomal protein L32* (RPL32) (35). The list of primers is provided in Table S1 in Supplementary Material.

### Protein Expression Profiling

Samples were processed using tissue protein extraction reagent (NE-PER kit) according to manufacturer’s protocol (Thermo Scientific). After centrifugation at 16,000 × *g* for 5 min, the protein in supernatant (Cytoplasmic extract) was transferred to prechilled tube. Insoluble fraction (pellet) was suspended in ice-cold nuclear extraction reagent, vortexed, centrifuged at 16,000 × *g* for 10 min. Supernatant (nuclear extract) was transferred to prechilled tubes. Protein was quantified by BCA method (Thermo Scientific). Nuclear protein extract was used for p65 subunit of NF-κB (p65 NF-κB) and NRF2 quantification while cytoplasmic protein extract was used for SOD2 protein quantification. TLR4 was measured from total protein fraction. Protein (30 µg) was resolved in 10% SDS-polyacrylamide gel using TGX stain free kit (Bio-Rad). After electrophoresis, protein was transferred to polyvinylidine difluoride membrane (GE Healthcare). Blocking of the membrane was performed using 3% non-fat milk in TBST containing 0.1% tween 20 at room temperature for 1 h, followed by appropriate primary antibody treatment overnight at 4°C. The membrane was washed with TBST for 5 min (three times). After washing, membrane was incubated with corresponding HRP-labeled secondary antibody at room temperature for 1 h. Membrane was washed with TBST (3 times for 5 min each) and the blot was visualized using gel doc XR system (Bio-Rad), using west dura pico (Thermo Scientific). Different antibodies used for the study are TLR4 (Abcam; Cat no. ab13444), p65 NF-kB (Abcam; Cat no. ab16502), oxidative phosphorylation (Abcam; Cat no. ab110413), NRF2 (Abcam; Cat no. ab31163), SOD2 (Abcam; Cat no. ab13533), anti-Rabbit, and anti-Mouse antibody.

### Statistical Analysis

All values are expressed as the mean ± SE. One-way analysis of variance test followed by Bonferroni’s correction was carried out to test for any differences between the mean values of all groups. Differences in group was assumed significant if *p* < 0.05.

## Results

### Effect of TLR4 Modulation on Heart Weight to Body Weight Ratio and Hypertrophy Markers

**Figure 1:**
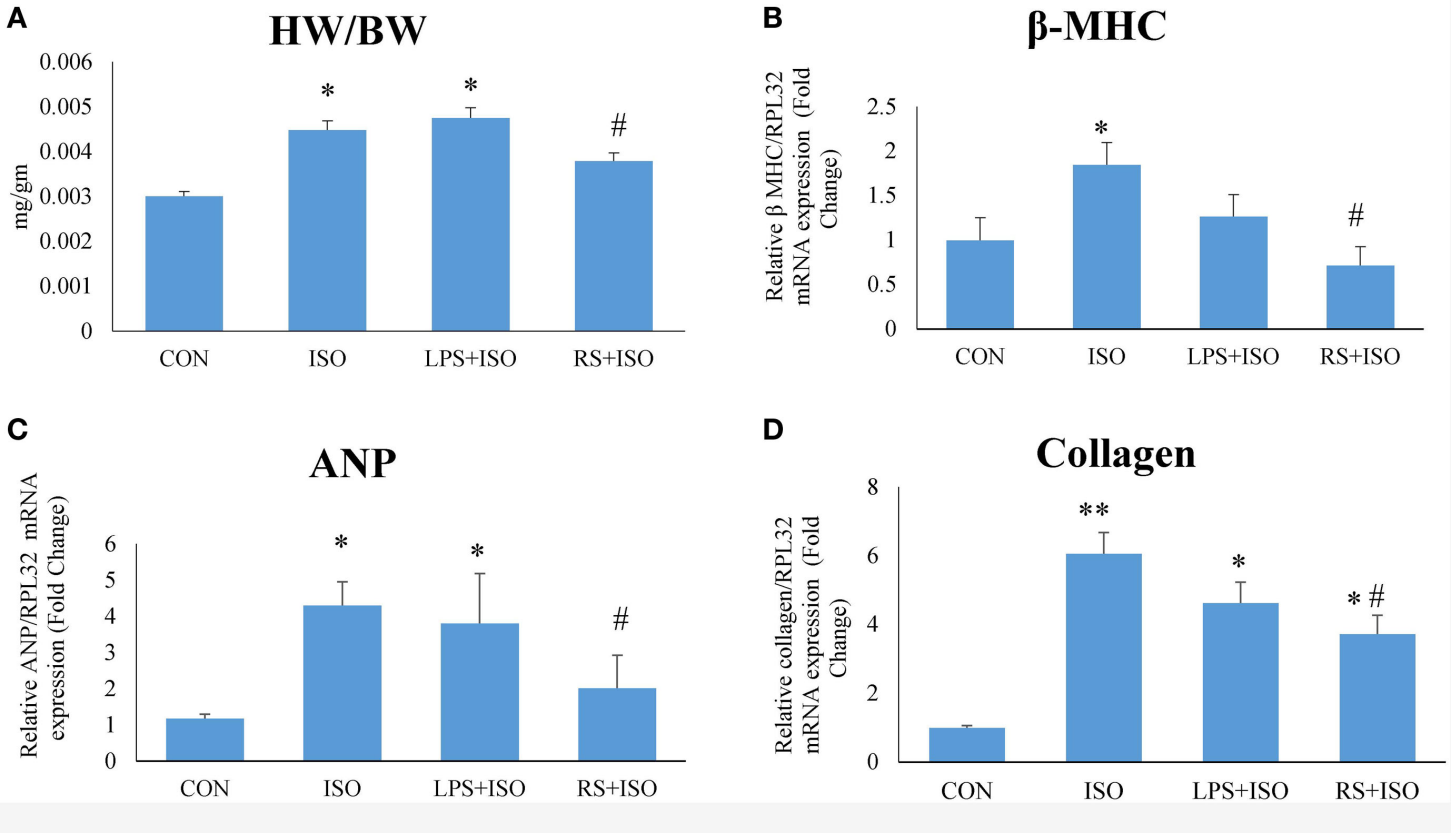
Cardiac phenotypes and mRNA expression of hypertrophy markers. **(A)** Heart weight to body weight ratio (HW/BW) **(B)** mRNA expression of beta MHC **(C)** mRNA expression of ANP **(D)** mRNA expression of collagen. The data were normalized to the expression of reference gene, *ribosomal protein L32* (RPL32). Data shown as mean ± SEM (*N* = 6 for HW/BW, *N* = 4 for mRNA expression) *p < 0.05, **p < 0.01 vs CON; ^#^*p* < 0.05, ^##^*p* < 0.01 vs ISO groups.

Heart weight to body weight ratio (Figure 1A), an indicator of cardiac hypertrophy, and mRNA expression of myocardial β*-*MHC (Figure 1B), ANP (Figure 1C), and collagen (Figure 1D) were increased significantly (*p* < 0.05) in ISO group as compared to CON group. RS treatment significantly (*p* < 0.05) decreased heart weight to body weight ratio and normalized the increased ANP, β*-*MHC, and collagen mRNA expression. However, LPS treatment did not restore the increased heart weight to body weight ratio and hypertrophy markers (Figure 1).

### TLR4 Inhibitor Decreased Cardiomyocyte Cell Size and Myocardial Fibrosis in Hypertrophy Heart

**Figure 2:**
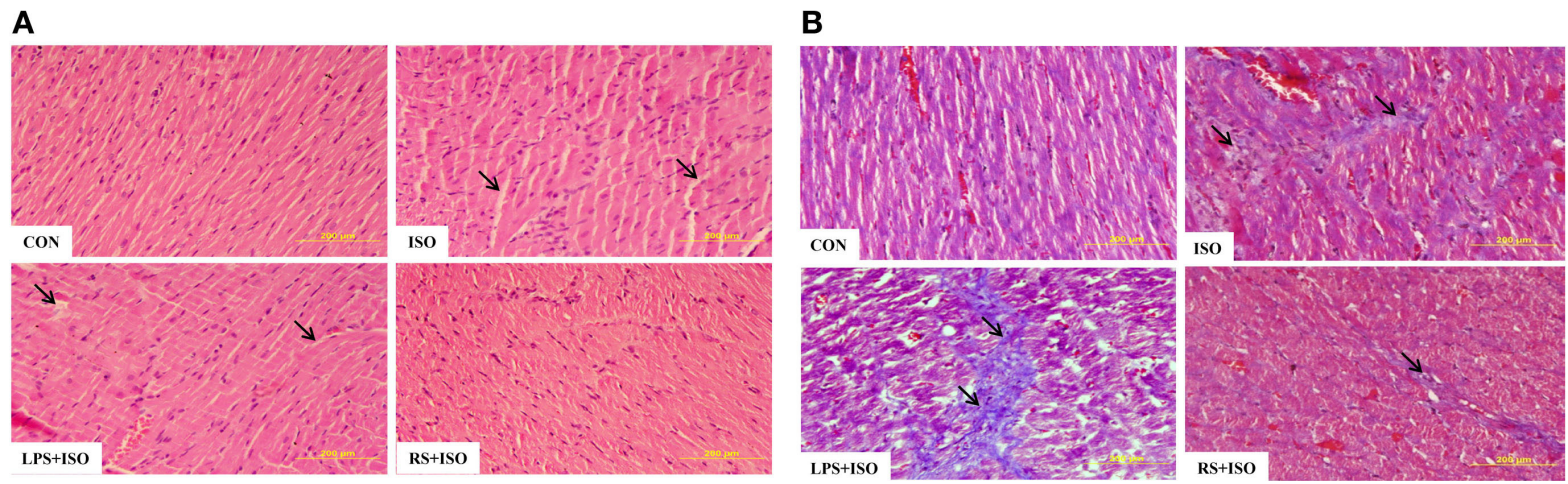
Cardiac cell phenotypes and histopathological examination in cardiac hypertrophy and effect of toll-like receptor 4 modulation. **(A)** Hematoxylin and eosin staining of rat heart tissue. Arrows in the figure represent loss of cardiomyocytes. **(B)** Masson’s trichrome staining of rat heart tissue. Arrows in figures represent the presence of fibrosis in heart tissue. Data shown as mean ± SEM, *p < 0.05, **p < 0.01 vs CON; ^#^*p* < 0.05, ^##^*p* < 0.01 vs ISO groups. Three sections were observed for histopathology examination and 20 cells per image were analyzed.

Cardiomyocyte size (Table S2 in Supplementary Material) and myocardial fibrosis (Figure 2; Figure S2 in Supplementary Material), which are hallmark of cardiac hypertrophy, were significantly (*p* < 0.05) increased in hypertrophy heart as compared to control. RS treatment significantly attenuated the increase in cardiomyocyte size and myocardial fibrosis (Figure 2B). LPS treatment did not change the ISO induced myocardial size but further increased myocardial fibrosis as compared to hypertrophy heart (Figure 2).

### TLR4 Inhibitor Improved Electrocardiograph Parameters

Electrocardiogram analysis showed prolonged QT interval (Figure S1C in Supplementary Material), increased R amplitude (Figure S1B in Supplementary Material) along with increased heart rate (Figure S1A in Supplementary Material) in ISO group. All these ECG changes in the hypertrophic heart indicate the presence of cardiac ventricular hypertrophy and tachycardia. RS (RS + ISO) administration in these rats normalized these altered electro cardiac abnormalities. LPS (LPS + ISO) treatment did not alter these perturbations in ECG (Figure S1 in Supplementary Material).

### TLR4 Expression Increased in Hypertrophy Heart

**Figure 3:**
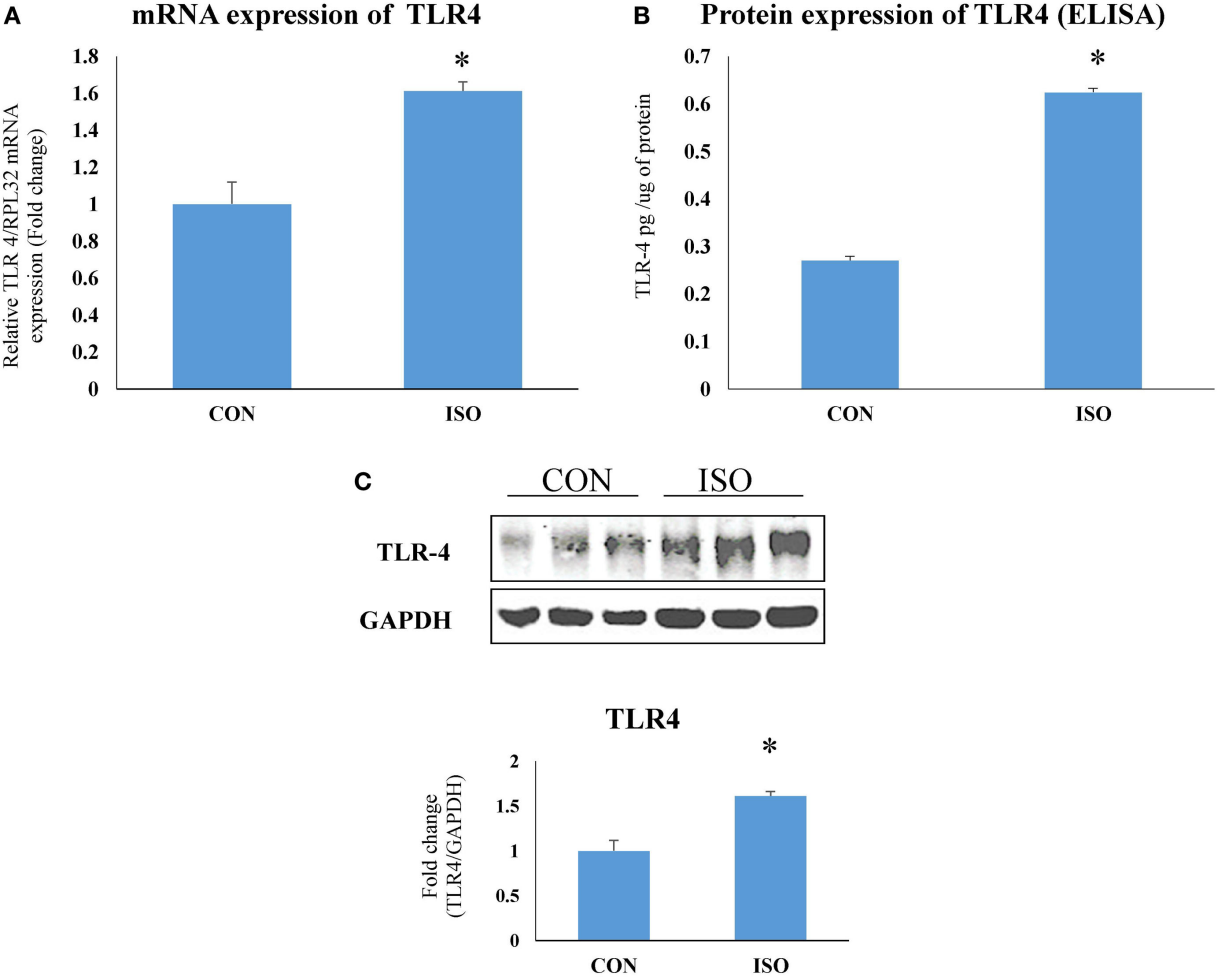
Toll-like receptor 4 (TLR4) expression was increased in cardiac hypertrophy. **(A)** mRNA expression. The data were normalized to the expression of reference gene, *ribosomal protein L32* (RPL32), **(B)** protein expression by ELISA, and **(C)** protein expression by western blot. The data were normalized to the expression of reference gene, GAPDH. Data shown as mean ± SEM (*N* = 3 for western blot and *N* = 4 for real-time PCR and ELISA) *p < 0.05, **p < 0.01 vs CON.

Toll-like receptor 4 mRNA (Figure 3A) and protein (Figures 3B,C) expression was significantly (*p* < 0.05) increased in rat hypertrophy heart as compared to control.

### TLR4 Inhibitor Attenuated Inflammatory Gene Expression in Hypertrophy Heart

**Figure 4:**
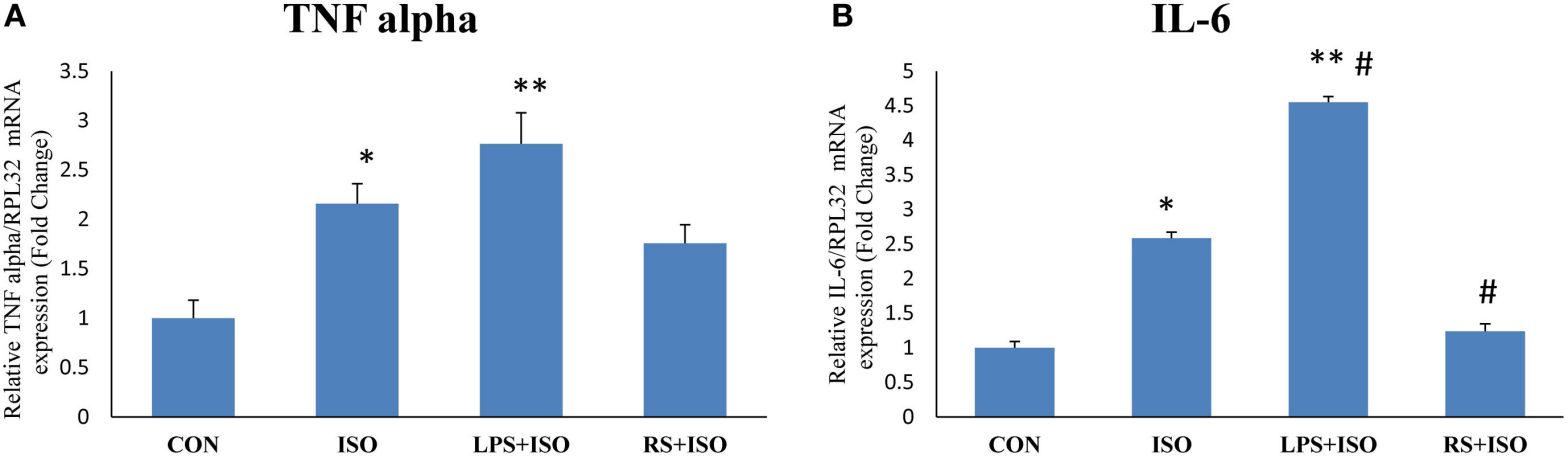
TNF alpha and IL-6 expression in hypertrophy rat heart after toll-like receptor 4 modulation. **(A)** mRNA expression of TNF alpha **(B)** mRNA expression of IL-6. The data were normalized to the expression of reference gene, *ribosomal protein L32* (RPL32). Data shown as mean ± SEM, *p < 0.05, **p < 0.01 vs CON; ^#^*p* < 0.05, ^##^*p* < 0.01 vs ISO groups.

Myocardial mRNA expression of inflammatory genes TNF alpha and IL-6 was significantly increased in ISO group as compared to CON group (Figures 4A,B). TLR4 inhibition decreased the mRNA expression of myocardial IL-6 (*p* < 0.05) as compared to ISO group. However, the level of TNF alpha was not significantly decreased by TLR4 inhibition as compared to ISO group (Figure 4). LPS treatment in ISO animals further increased the level of IL-6 expression (*p* < 0.05).

### TLR4 Inhibitor Reduced Cardiac Oxidative Stress in Hypertrophy Heart

**Figure 5:**
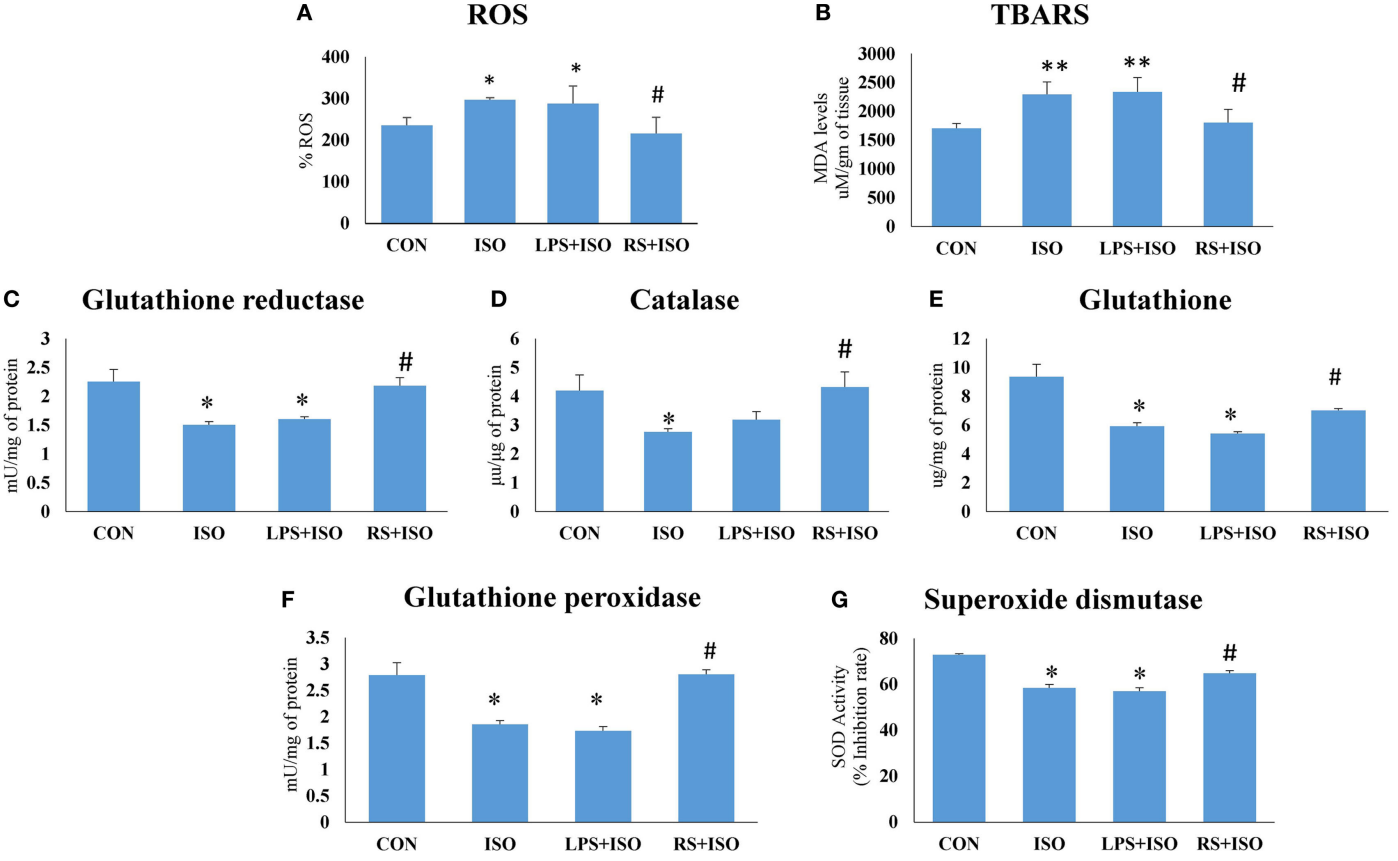
Cardiac redox status in hypertrophic condition and effect of toll-like receptor 4 modulation on myocardial **(A)** reactive oxygen species (ROS), **(B)** thiobarbituric acid reactive substances (TBARS), **(C)** glutathione reductase activity, **(D)** catalase activity, **(E)** reduced glutathione, **(F)** glutathione peroxidase activity, and **(G)** superoxide dismutase activity. Data shown as mean ± SEM (*N* = 5) *p < 0.05, **p < 0.01 vs CON; ^#^*p* < 0.05, ^##^*p* < 0.01 vs ISO groups.

There was a significant (*p* < 0.05) increase in cardiac ROS (Figure 5A), and TBARS level (Figure 5B), and decrease (*p* < 0.05) in cardiac endogenous antioxidants like GR (Figure 5C), CAT (Figure 5D), GSH (Figure 5E), GPx (Figure 5F), and SOD (Figure 5G) in ISO group. RS treatment decreased (*p* < 0.05) cardiac ROS and TBARS level and increased endogenous antioxidants toward normal. However, we have not observed any improvement in disturbed redox balance after LPS treatment (Figure 5).

### TLR4 Inhibitor Improved Mitochondrial ETC Complex Function in Hypertrophy Heart

**Figure 6:**
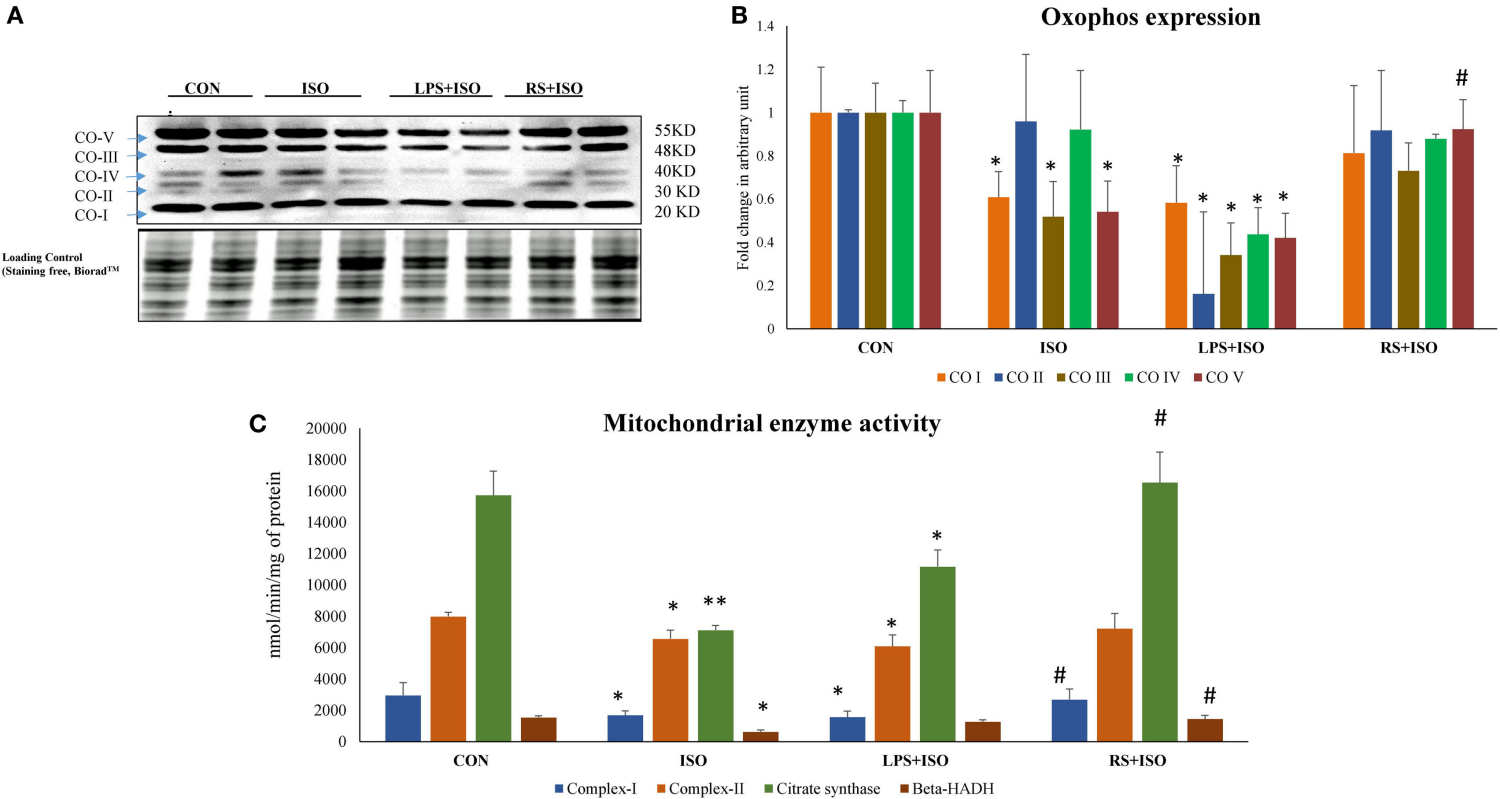
Protein expression of mitochondrial complexes and their activity in hypertrophic rat heart and effect of toll-like receptor 4 (TLR4) modulation. **(A)** Protein expression of mitochondrial complexes, **(B)** fold change in expression of mitochondrial complexes (I–V) in all study groups. Whole gel stain was used for loading control, **(C)** mitochondrial metabolic enzymes activity in hypertrophic rat heart and effect of TLR4 modulation. (I) complex-I: NADH dehydrogenase, (II) complex-II: succinate dehydrogenase, (III) citrate synthase (IV) β-hydroxy acylcoA dehydrogenase. Data shown as mean ± SEM (*N* = 4 for activity and *N* = 3 for western blot) *p < 0.05, **p < 0.01 vs Con; ^#^*p* < 0.05, ^##^*p* < 0.01 vs ISO groups.

Protein expression of mitochondrial complex I, III, and V (Figures 6A,B) was significantly (*p* < 0.05) reduced in ISO group. RS treatment restored the decreased protein expression of these mitochondrial complexes toward normal. LPS treatment found to further decrease the protein expression of these complexes in ISO group (*p* < 0.05) (Figure 6).

### TLR4 Inhibitor Improves Mitochondrial Enzyme Activities in Hypertrophy Heart

Myocardial citrate synthase (Figure 6C) and 3-hydroxy-CoA dehydrogenase (Figure 6C) activity was significantly (*p* < 0.05) reduced in hypertrophic animals. RS treatment in ISO animals significantly (*p* < 0.05) increased citrate synthase and 3-hydroxy-CoA dehydrogenase activity. LPS treatment failed to restore the detrimental perturbations in these enzyme activities. Protein expression results of ETC complexes correlated directly with the enzyme activity. Myocardial enzyme activity of mitochondrial complex I, i.e., NADH dehydrogenase (Figure 6C) and complex II, i.e., succinate dehydrogenase (Figure 6C) were reduced in ISO animals as compared to CON group. However, these activities were normalized by RS treatment (*p* < 0.05). LPS treatment failed to increase the decreased enzyme activity of these enzymes (Figure 6).

### TLR4 Inhibitor Attenuated Myocardial p65 NF-κB and NRF2 and Restored MnSOD Expression in Hypertrophy Heart

**Figure 7:**
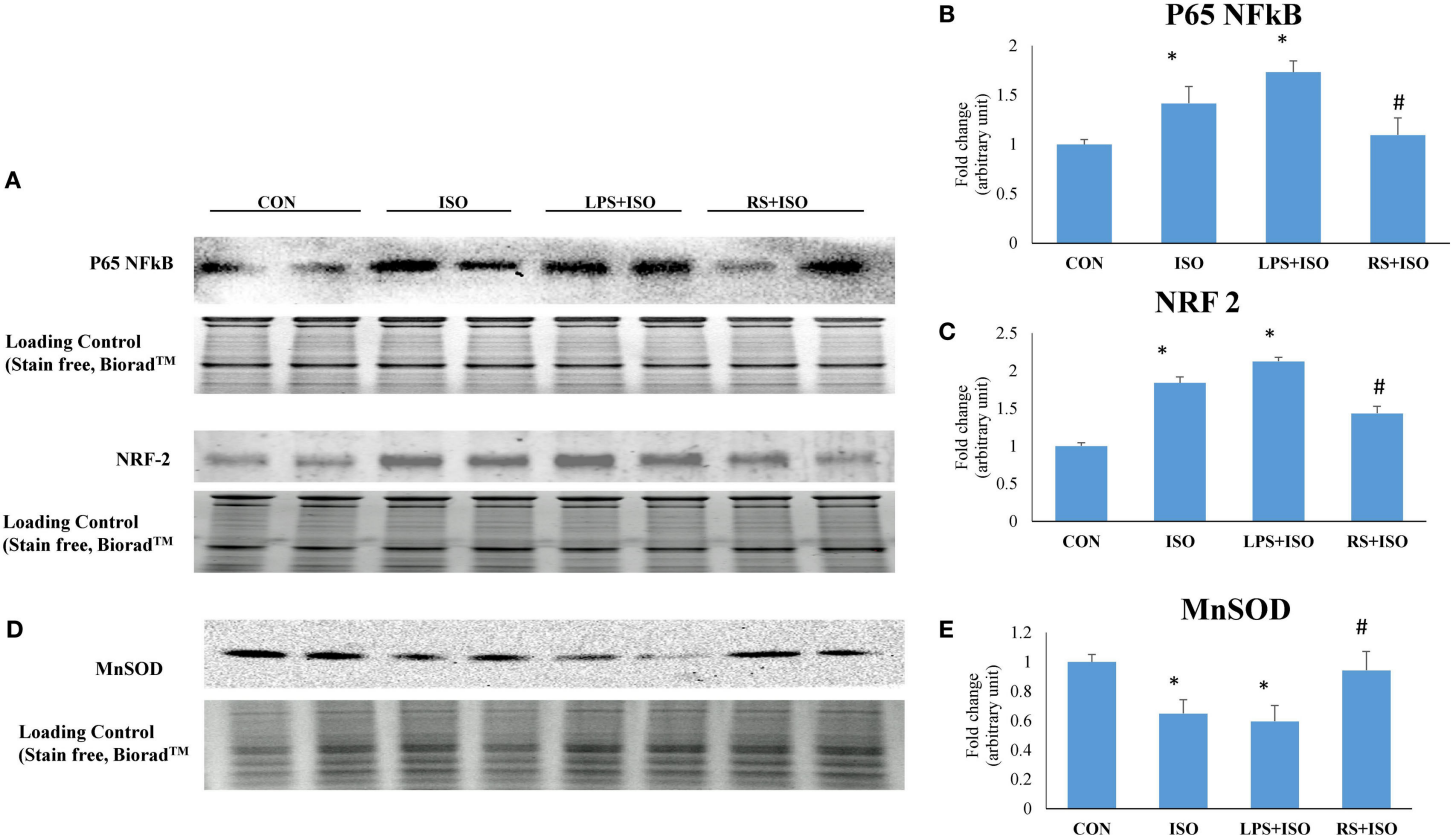
Protein expression of p65 NF-κB, NRF2, and MnSOD in hypertrophic rat heart and effect of toll-like receptor 4 modulation. **(A)** Protein expression of p65 NF-κB and NRF2, **(B)** fold change in p65 NF-κB protein expression, **(C)** fold change in NRF2 protein expression, **(D)** protein expression of MnSOD, **(E)** fold change in MnSOD protein expression. Whole gel stain was used for loading control. Data shown as mean ± SEM (*N* = 3) *p < 0.05, **p < 0.01 vs CON; ^#^*p* < 0.05, ^##^*p* < 0.01 vs ISO groups.

Myocardial p65 NF-κB and NRF2 protein expression was significantly increased in ISO group. LPS treatment further increased the expression of p65 NF-κB in hypertrophy heart. However, RS treatment reduced the increased level of P65 NF-κB and NRF2 in ISO treated rat heart (Figures 7A–C). Myocardial MnSOD level was significantly down in ISO animals (*p* < 0.05). LPS treatment leads to further decrease in the MnSOD level. However, RS treatment restored the decreased level of MnSOD toward normal (*p* < 0.05) (Figures 7D,E).

The animals with only RS-LPS treatment did not show any changes in normal physiology, whereas LPS-treated animals shown an increased cardiac fibrosis and increased oxidative stress (data not shown).

## Discussion

The concept that innate immunity may constitute a component of “adaptive cardiac biology” is being increasingly recognized (36). The components of the innate immunity program seem to be required to resist infectious and pressure-overload-mediated cardiac decompensation and heart failure. PRRs, part of innate immunity, play an important role in the identification of danger signals released from diseased heart (37). DAMPs released from injured cardiomyocytes acts as a danger signal (38). Recent literature shows that DAMPs released from the necrotic heart tissue alone are sufficient to induce chronic myocardial inflammation (39). Binding of DAMPs to PPRs on the cell surface leads to signaling *via* NF-κB and MAP kinase pathways leading to pro-inflammatory cytokine expression (IL-1, TNF-α, IL-12), chemokine secretion (IL-8, monocyte chemotactic protein-1), and thus leukocyte infiltration and inflammation (40). Recently, many studies have shown that TLR4 plays an important role in the cardiac adaptation during decompensated state of the system (41). LPS is a proven TLR4 agonist. *In vivo*, LPS activates innate immune system and evokes inflammatory responses (36). A structural analog of LPS, RS-LPS (RS), a well-known TLR4 inhibitor, has shown anti-inflammatory responses in the body. While TLR4 inhibitor may attenuate hypertrophy through inhibition of signaling pathway of TLR4, mild activation of TLR4 before injury may activate adaptive response in heart (42). Therefore, in the present study, we proposed to find the effect of TLR4 modulation on hypertrophied heart.

Toll-like receptor 4 was observed to be perturbed in many cardiac diseases including diabetic and hypertrophic cardiomyopathy (43). This dysregulation in the expression of TLR4 suggests that intact immunity of the heart is deliberately imbalanced, which may lead to increased response toward DAMPs. Protein and gene expression of TLR4 was significantly increased in cardiac hypertrophy. In the present study, we used isoproterenol-induced cardiac hypertrophy model as used in previous studies (44, 45). Isoproterenol is a sympathetic beta receptor agonist, and when administered parenterally, mimics catecholamine’s action on heart. Catecholamine-induced cardiac hypertrophy is a class of “stress induced or anxiety induced hypertrophy” (46). Stress and anxiety leads to increased catecholamine’s in body. Prolonged exposure to high level of catecholamine’s leads to cardiac hypertrophy (47). This model is more correlated with today’s stressful lifestyle. Similar to other studies, we found increased heart weight/body weight ratio, gene expression of ANP, collagen, and beta MHC, which are the markers of cardiac hypertrophy along with increased cardiomyocytes size in cardiac hypertrophy. Administration of TLR4 inhibitor (RS) attenuated most of these hypertrophy changes in heart. RS treatment in hypertrophy animals proved to be effective to revert the changes in cardiac system caused by isoproterenol. This improved cardiac health was also supported by ECG analysis. ISO administration in rats increased QT interval as well as R wave amplitude, which are the indications of left ventricular hypertrophy (48). These perturbations were restored toward normal with RS treatment. Whereas, TLR4 agonist (LPS + ISO) does not show any improvement in hypertrophy markers and ECG parameters. This indicates that TLR4 inhibitor could terminate the cardiac hypertrophy complications and its progression. However, there was no improvement of all these parameters in TLR4 agonist (LPS)-treated hypertrophy heart. Increased cardiac inflammation was successfully attenuated with RS treatment as indicated by IL-6 gene expression. LPS-treated hearts were exposed to higher level of inflammation as compared to ISO- and RS-treated hearts.

Cardiac hypertrophy is often associated with alteration of cardiomyocyte redox balance. To analyze the redox system in all groups, we have measured myocardial lipid peroxidation and endogenous antioxidants like SOD, catalase, GR, and GPx activity and reduced glutathione (GSH) levels. Cardiac lipid peroxidation was found to be increased and activity of all antioxidant enzymes was decreased in ISO heart as compared to CON. RS treatment in ISO animals prevented these detrimental perturbations in redox system. However, LPS did not restore the disturbed redox balance in ISO animals.

We then assessed mitochondrial health to find out the source of redox imbalance and found wide perturbations in the ISO group, which were ameliorated with RS treatment. To confirm the role of TLR4 on mitochondrial function in cardiac hypertrophy, we have analyzed the protein expression of ETC complexes in heart of these animals. Protein expression of complex-I, III, and V was significantly decreased in ISO heart as compared to control. LPS treatment in ISO animals further reduced the mitochondrial protein expression of these complexes. RS treatment in ISO animals (RS + ISO) retained the mitochondrial complexes protein expression to normal as compared to ISO animals. Our data also suggest that mitochondrial enzyme activity is the direct outcome of TLR4 modulation. The activity of complex I and II, citrate synthase, an important enzyme from TCA cycle and 3-hydroxyacyl-CoA dehydrogenase from beta-oxidation pathway in hearts were found to be significantly decreased in ISO animals. RS treatment in hypertrophy animals (RS + ISO) preserved the activity of these enzymes. However, data suggest that LPS (TLR4 agonist) treatment did not improve the mitochondrial health and function of ETC in ISO heart and even aggravated the dysfunction in few instances. This shows the role of TLR4 in regulating mitochondrial function, which is supported with previous literature (49, 50).

Myocardial protein expression of p65 NF-κB and NRF2 were significantly increased in ISO heart. Although increased NFκB signifies increased inflammatory response in cardiomyocytes, increased level of NRF2 may signify a state of cell where defense mechanism initiated to overcome increased oxidative stress. RS treatment attenuated the oxidative stress in the cell and hence the expression of p65 NF-κB and NRF2. This indicates decreased induction of inflammation in heart due to decreased activation of TLR4. Whereas, LPS further increased the p65 NF-κB and NRF2 expression indicating further enhanced inflammation in heart. In heart, SOD is the primary defense against ROS (51). Approximately 70% SOD activity in the heart and 90% that in cardiomyocytes is contributed by MnSOD (52). The reduced level of MnSOD in hypertrophy heart is the indication of increased mitochondrial oxidative stress. Mitochondrial oxidative stress was further increased with LPS treatment in hypertrophy heart. RS treatment successfully restored decreased level of MnSOD in mitochondria. This decrease in oxidative stress could be one of the reason behind improved mitochondrial health after RS treatment in hypertrophy heart.

In general, cellular damage that occurs during stress and tissue injuries is often accompanied with mitochondrial dysfunction, followed by cellular apoptosis associated with release of mitochondria-derived components (42, 53, 54). It is clear that certain amount of tissue apoptosis and cellular factors turnover occurs during normal physiology. However, the amount of TLR4 ligands pool in such cases is most likely to be below the threshold level to initiate an inflammatory cascade (55). However, during pathological state like mitochondrial dysfunction, mitochondrion releases high-affinity endogenous TLR4 ligands and induces TLR4-mediated inflammation (56). Oxidative stress induced cardiac injury, a result of mitochondrial dysfunction, may lead to the release of these endogenous TLR4 ligands that bind with TLR4 and initiate the inflammatory cascade. This increased TLR4 activation due to DAMP released from mitochondria may lead to excessive cardiomyocyte damage in a state of cardiac hypertrophy.

## Conclusion

We found increased cardiac TLR4 expression along with increased oxidative stress and mitochondrial dysfunction in hypertrophy heart. We have demonstrated that having increased TLR4 ligand pool (LPS) during cardiac hypertrophy may accelerate the disease progression and aggravate the disease agony. Our data showed that inhibition of TLR4 in hypertrophy group attenuated cardiac hypertrophy through restoring cardiac redox balance and mitochondrial dysfunction. Further studies are required to connect the missing link between the role of TLR4 in mitochondrial dysfunction and cardiac hypertrophy.

## Ethics Statement

All experiments involving animals were undertaken with the approval of Institutional Animal Ethical Committee of Indian Institute of Chemical Technology, Hyderabad.

## Author Contributions

PK and PB carried out animal experimentation, biochemical and molecular estimation, and statistical analysis of results. AD did the histopathological examination of heart tissue. PK and SB conceived the study, and participated in its design, coordination, and drafted the manuscript. The authors read and approved the manuscript.

## Conflict of Interest Statement

The authors declare that the research was conducted in the absence of any commercial or financial relationships that could be construed as a potential conflict of interest.

## Abbreviations

TLR4, toll-like receptor 4; PRRs, pattern recognition receptors; OXPHOS, oxidative phosphorylation; PAMPs, pathogen-associated molecular patterns; DAMP, damage-associated molecular pattern; ROS, reactive oxygen species.
