# Supplementary material for: Toll-Like Receptor 4 Inhibition Improves Oxidative Stress and Mitochondrial Health in Isoproterenol-Induced Cardiac Hypertrophy in Rats
Source: Front Immunol. 2017 Jun 22;8:719. doi: 10.3389/fimmu.2017.00719 (PMC5479928; doi:10.3389/fimmu.2017.00719)
Supplement: Supplementary file 1 [file Data_Sheet_1.DOCX]

| Gene name | Forward primer | Reverse primer |
| --- | --- | --- |
| TLR 4 | \| \| TTGCTGCCAACATCATCCAG \| \| --- \| \| \| --- \| --- \| | \| \| TACAATTCGACCTGCTGCCT \| \| --- \| \| \| --- \| --- \| |
| ANP | AGCGAGCAGACCGATGAAG | AGCCCTCAGTTTGCTTTTCA |
| RPL32 | AGATTCAAGGGCCAGATCCT | CGATGGCTTTTCGGTTCTTA |
| Collagen | CTCAAGAGCGGAGAATAC | ATCTGTCCACCAGTGCTT |
| IL 6 | \| \|  AGGACCAAGACCATCCAAC \| \| --- \| \| \| --- \| --- \| | \| \| ACCACAGTGAGGAATGTCCA \| \| --- \| \| \| --- \| --- \| |
| TNF Α | CGACTCTGACCCCCATTACT | CGTCTCGTGTGTTTCTGAGC |
| β MHC | TGGAGCTGATGCACCTGTAG | ACTTCGTCTCATTGGGGATG |

**Table S1.** List of primers used in the gene expression profiling.


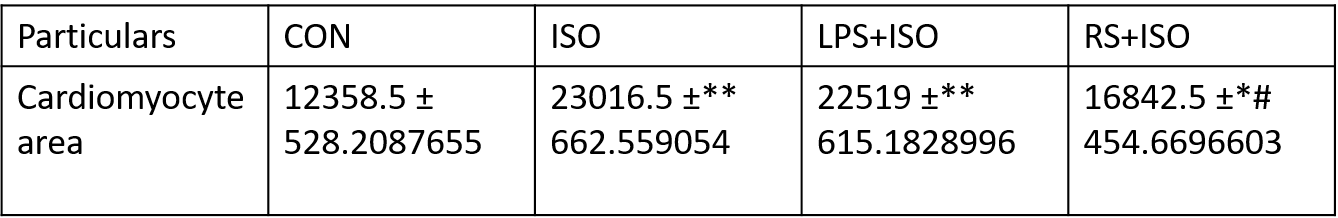


**Table S2.** Cardiomyocyte area in hypertrophy rat heart after TLR4 modulation.

Data shown as Mean ± SEM, *p<0.05, **p<0.01 vs CON; #p<0.05, ##p<0.01 vs ISO groups.


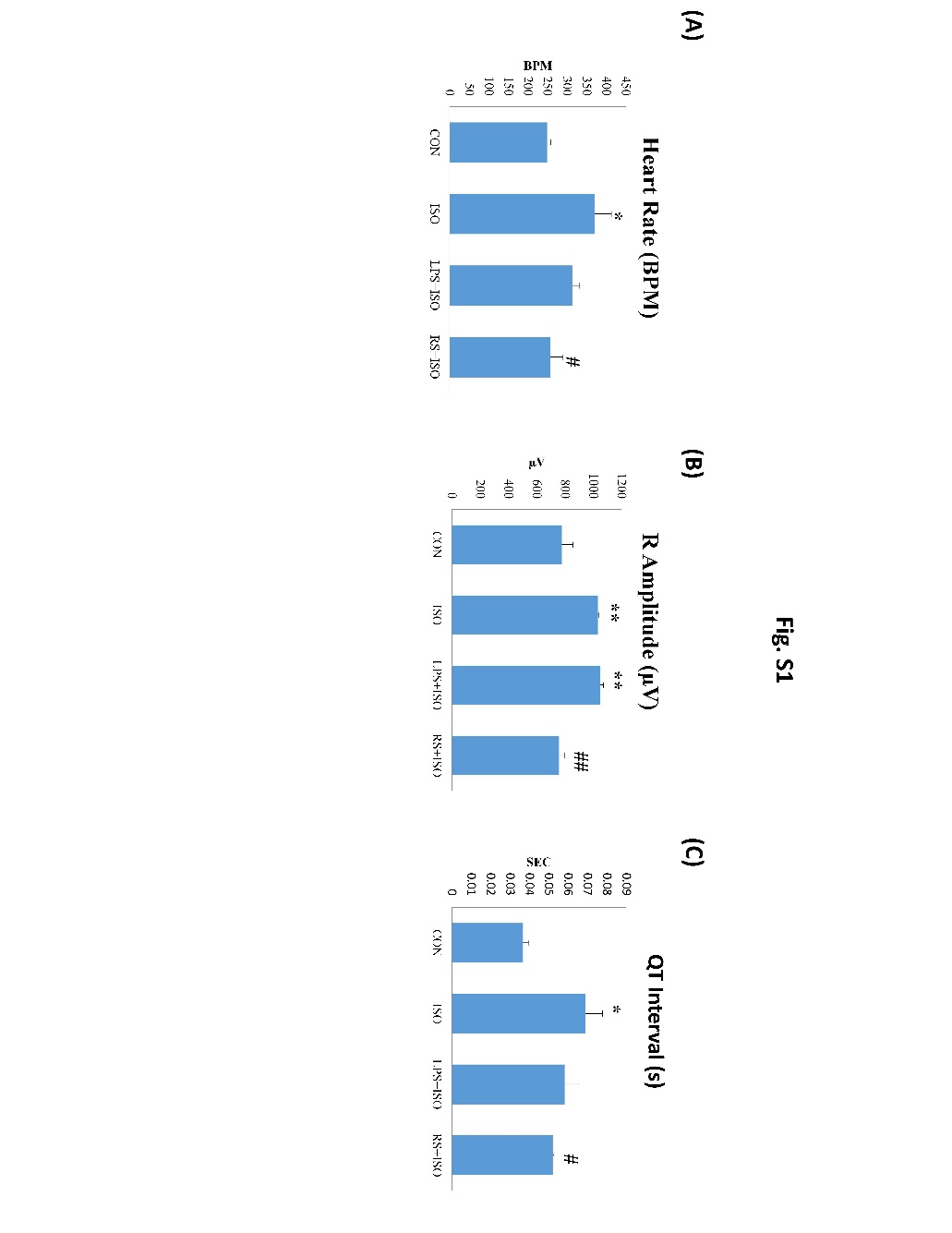


**Fig S1.** Electrocardiogram (ECG) perturbations in cardiac hypertrophy and effect of TLR4 modulation. (A) heart rate in beats per minute (BPM) (B) R amplitude (C) QT duration. Data shown as Mean ± SEM, (N=4) *p<0.05, **p<0.01 vs CON; #p<0.05, ##p<0.01 vs ISO groups.

**#**

****##**

**Fig S2.** Cardiac fibrosis in hypertrophic condition and effect of TLR4 modulation. Data shown as Mean ± SEM, *p<0.05, **p<0.01 vs CON; #p<0.05, ##p<0.01 vs ISO groups.
